# Neural Activation *via* Acupuncture in Patients With Major Depressive Disorder: A Functional Near-Infrared Spectroscopy Study

**DOI:** 10.3389/fpsyt.2021.669533

**Published:** 2021-11-12

**Authors:** Tingyu Zhang, Jiaqi Zhang, Jiaxi Huang, Zhong Zheng, Pu Wang

**Affiliations:** ^1^Department of Rehabilitation Medicine, The Seventh Affiliated Hospital Sun Yat-sen University, Shenzhen, China; ^2^Department of Rehabilitation Sciences, The Hong Kong Polytechnic University, Kowloon, Hong Kong SAR, China; ^3^Mental Health Center, West China Hospital/West China School of Medicine, Sichuan University, Chengdu, China; ^4^Guangdong Engineering and Technology Research Center for Rehabilitation Medicine and Translation, Guangzhou, China

**Keywords:** major depressive disorder, acupuncture, pre-frontal cortex, neuroplasticity, functional near-infrared spectroscopy (fNIRS)

## Abstract

**Background and Objective:** Acupuncture is used as an alternative treatment for patients with major depressive disorder (MDD). The associated therapeutic effect of acupuncture is often attributed to its modulatory effect on the activity of the pre-frontal cortex (PFC), although the mechanism is not well-studied. We employed a repeated measures design to investigate the brain modulatory effect of acupuncture on the PFC in a group of patients with MDD and investigated whether the modulatory effect is influenced by the severity of the disease.

**Methods:** A total of 47 patients diagnosed with MDD were enrolled in this functional near-infrared spectroscopy experiment. The severity of depressive symptoms was measured at baseline using the Hamilton Depression Rating Scale-24 (HAMD). The cortical activation in the bilateral PFC areas during a verbal fluency task (VFT) was measured before and after a single session of acupuncture in the Baihui acupoint. We further explored the potential correlation between the severity of MDD and task-related activation before and after acupuncture.

**Results:** A single session of acupuncture significantly tended to enhance the activation level of the left frontopolar cortex in patients with severe depression during VFT, but a null effect was found in those with mild to moderate depression. Among patients with severe depression, a strong correlation was observed between HAMD scores and the change in VFT-related activation after acupuncture in the left dorsolateral PFC (DLPFC).

**Conclusion:** A single session of acupuncture did not significantly modulate the activation of the left PFC in patients with mild to moderate depression; however, it demonstrated a tendency to enhance the activation of the frontopolar area in patients with severe depression. Among patients with severe depression, there is a correlation between the activation by acupuncture of left DLPFC during executive functioning and the severity of depressive symptoms, suggesting that the brain activity induced by acupuncture is likely to be influenced by the baseline disease severity in patients with MDD.

## Introduction

Patients with major depressive disorder (MDD) present with impaired pre-frontal cortex (PFC) functioning, that is, decreased cerebral blood flow (1) and glucose hypometabolism (2) in the PFC during either resting or task conditions. The level of reduced neural activation is correlated with the severity of the disease (3). The PFC is implicated in cognitive processing and emotion regulation (4), which explains the cognitive and emotional deficits associated with MDD. Severe depression is associated with serious consequences, such as suicidal ideation and behaviors (5). Therefore, there is a need to develop novel treatment approaches for MDD and conduct mechanistic studies to scientifically underpin the efficacy of those treatments.

Acupuncture, a therapeutic modality of traditional Chinese medicine (TCM), has been applied as an alternative treatment for the regulation of cognition and emotion in patients with MDD. Acupuncture is characterized by the insertion of thin needles into acupoints on the human body. The needle is twirled several times until the patients feel a sensation of “de-qi,” which literally means “the arrival of vital energy” (6). The dysregulation caused by MDD can be explained by the concept of “Yu,” which refers to “mental constraint” (7). According to a recent meta-analysis, acupuncture is a useful treatment modality to reduce the severity of depressive symptoms, with a moderate effect size (8). The acupoint Baihui (GV20), which belongs to the Governor Vessel, is positioned at the highest point on the head where all the Yang meridians converge. Acupuncture in Baihui is widely used by TCM practitioners to treat neuropsychiatric disorders, because it is believed to be the key point that can regulate the “qi” of the Governor Vessel and release mental constraints. The activation of the PFC induced by Baihui electro-acupuncture has also been previously reported (9). Antidepressant treatments lead to a reduction in depressive symptoms and are often associated with increasing PFC activity during cognitive tasks (2, 10). Enhanced levels of PFC activity are also linked with the therapeutic effects of excitability-enhancing brain stimulation in patients with depression (11). Therefore, there is a possibility that the modulatory effect of Baihui acupuncture on the activation of the PFC can, at least partly, contribute to its therapeutic effects on MDD. However, the modulatory effect of Baihui acupuncture on the activation of the PFC has not yet been well-established. Functional near-infrared spectroscopy (fNIRS) has become a popular brain imaging technique. With fair temporal and spatial resolutions, fNIRS is a suitable neuroimaging modality for investigating regional neural dynamics associated with the whole process of acupuncture manipulation (i.e., needle insertion, twirls, and removal) (12). In this study, we first conducted an fNIRS experiment to reveal the modulatory effects of Baihui acupuncture on the PFC among a group of individuals diagnosed with MDD. Besides, according to previous literature, the activity of the PFC is likely to be correlated with the severity of MDD symptoms (13); therefore, we then explored the potential relationship between the severity of MDD and activation response to acupuncture. We hypothesized that (1) a single session of Baihui acupuncture could increase the activation of the PFC during executive functioning in patients with depression, and (2) the activation response to acupuncture could be influenced by the baseline severity of depressive symptoms.

## Methods

### Participants

A total of 47 patients (mean age = 39.70 ± 12.24 years, 37 females) who met the criteria for MDD of the Diagnostic and Statistical Manual of Mental Disorders, fourth edition, were consecutively enrolled from the West China Hospital outpatient clinic. The patients were invited to participate in this experiment if they met all of the following inclusion criteria: (1) aged between 18 and 60 years; (2) right-handed, as assessed by the Edinburgh Handedness Inventory (EDI) [patients whose EDI laterality quotient, that is (right–left)/(right + left), was >0.4 were considered as right-hand dominant] (14); (3) severity of depressive symptoms score ≥8 according to the Hamilton Depression Rating Scale (HAMD); and (4) having >6 years of formal education. Patients were excluded if they met any of the following exclusion criteria: (1) any known mental disorder excluding MDD, such as schizophrenia, substance use disorder, or obsessive-compulsive disorder; (2) serious cardiovascular or neurological diseases; (3) intellectual disabilities or language or hearing impairments; or (4) pregnancy. Patients were requested to provide written informed consent before their enrollment. The demographic and clinical details of the patients who participated in the study are presented in Table 1. The study was approved by the Human Research Ethics Committee of West China Hospital (Ethics approval number: WCH201801201134).

**Table 1:** Demographic and clinical characteristics of included participants.

**Participant characteristic**	**Participants** **(***n*** = 47)**	**Mild to moderate** **depression (***n*** = 33)**	**Severe depression** **(***n*** = 14)**	**Between-group** **difference^#^ ***p*****
**Demographic**				
Age (years, mean ± SD)	39.70 ± 12.24	42.21 ± 11.63	33.79 ± 11.99	0.029*
Male/female	10/37	9/24	1/13	0.242
Education (years, mean ± SD)	14.32 ± 3.07	14.12 ± 2.77	14.79 ± 3.75	0.560
**Clinical**				
Number of depressive episodes (mean ± SD)	1.94 ± 1.12	1.85 ± 0.97	2.20 ± 1.48	0.715
Age at disease onset (years, mean ± SD)	34.66 ± 13.60	37.00 ± 13.17	29.14 ± 13.40	0.071
Duration of disease (years, mean ± SD)	5.04 ± 6.58	5.21 ± 7.36	4.64 ± 4.43	0.620
Number of antidepressant medications in current episode, % (*n*)				
None	63.83% (30)	78.57% (19)	57.58% (11)	0.299
One	25.53% (12)	14.29% (10)	30.30% (2)	
Two	8.51% (5)	7.14% (4)	12.12% (1)	
HAMD-24 scores (mean ± SD)	29.87 ± 10.48	24.30 ± 6.47	43.00 ± 4.64	<0.001***

*SD, standard deviation; HAMD-24, 24-item Hamilton Depression Scale*.

*Antidepressant medications included selective serotonin reuptake inhibitors and serotonin-norepinephrine reuptake inhibitors*.

# *Comparisons between patients with mild to moderate and severe depression*.

* *p < 0.05*;

*** *p < 0.001*.

### Procedures

The entire experiment comprised three stages: a 160-s verbal fluency task (VFT) before the acupuncture session (pre-acu), a 220-s acupuncture manipulation, and a 160-s VFT after the acupuncture session (post-acu).

#### Verbal Fluency Task

A Chinese-language phonological VFT was utilized in this study. The task required patients to generate words beginning with a given Chinese character. VFTs are the most widely used and reliable paradigm to elicit activity in the PFC (15–17). Neural activity during VFTs has also been proposed as a surrogate biomarker to differentiate patients with MDD from healthy controls (18).

**Figure 1:**
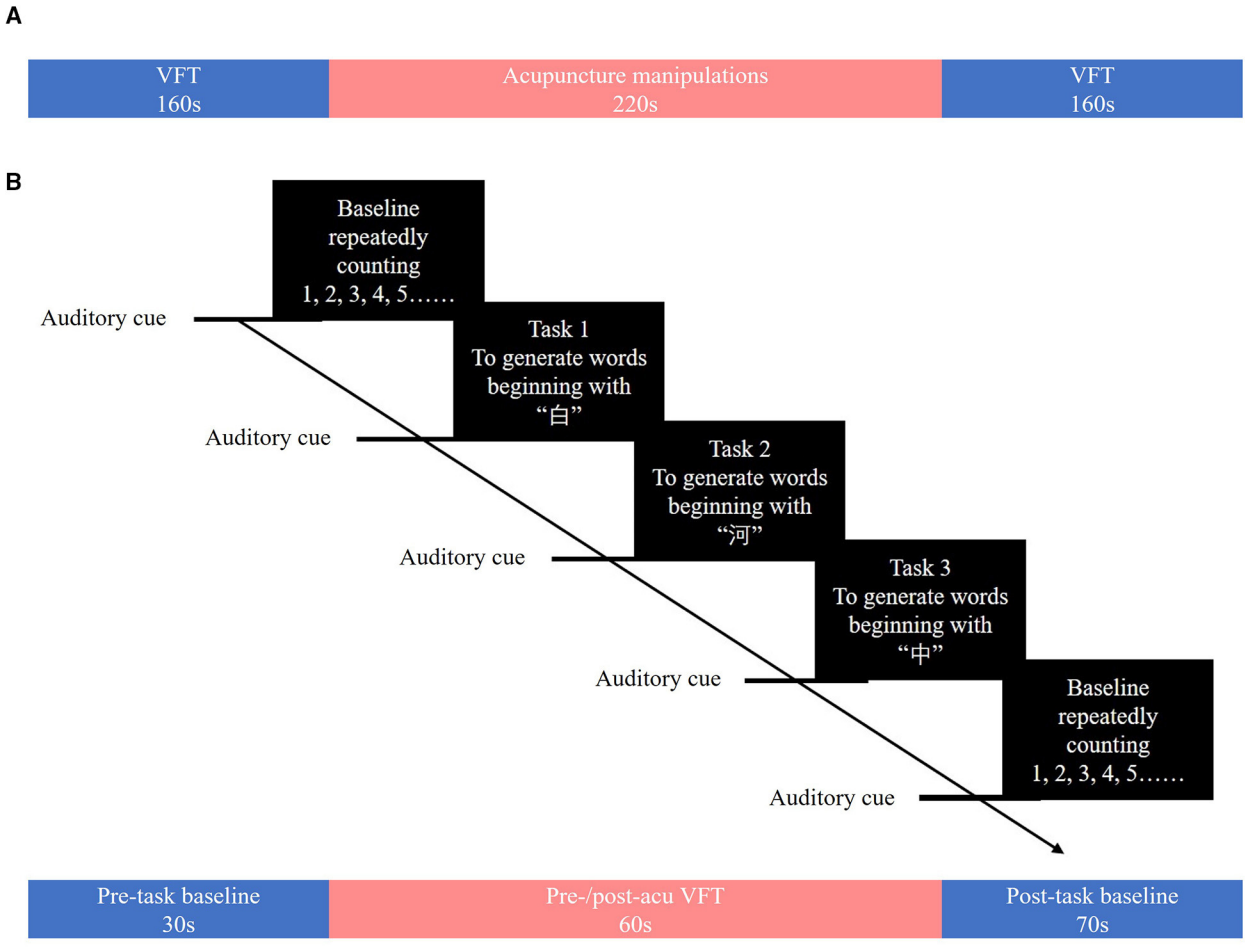
**(A)** Overview of the study. **(B)** The verbal fluency task. The verbal fluency task consisted of a 30-s pre-task resting-state period and three 20-s word-generating tasks followed by a 70-s post-task resting-state period.

The 160-s block-designed VFT, which was used in this study, consisted of a 30-s pre-task period, three consecutive blocks of 20-s word-generating tasks, and a 70-s post-task period (Figure 1). In the pre- and post-task periods, patients were asked to repeatedly count from one to five aloud.

**Figure 2:**
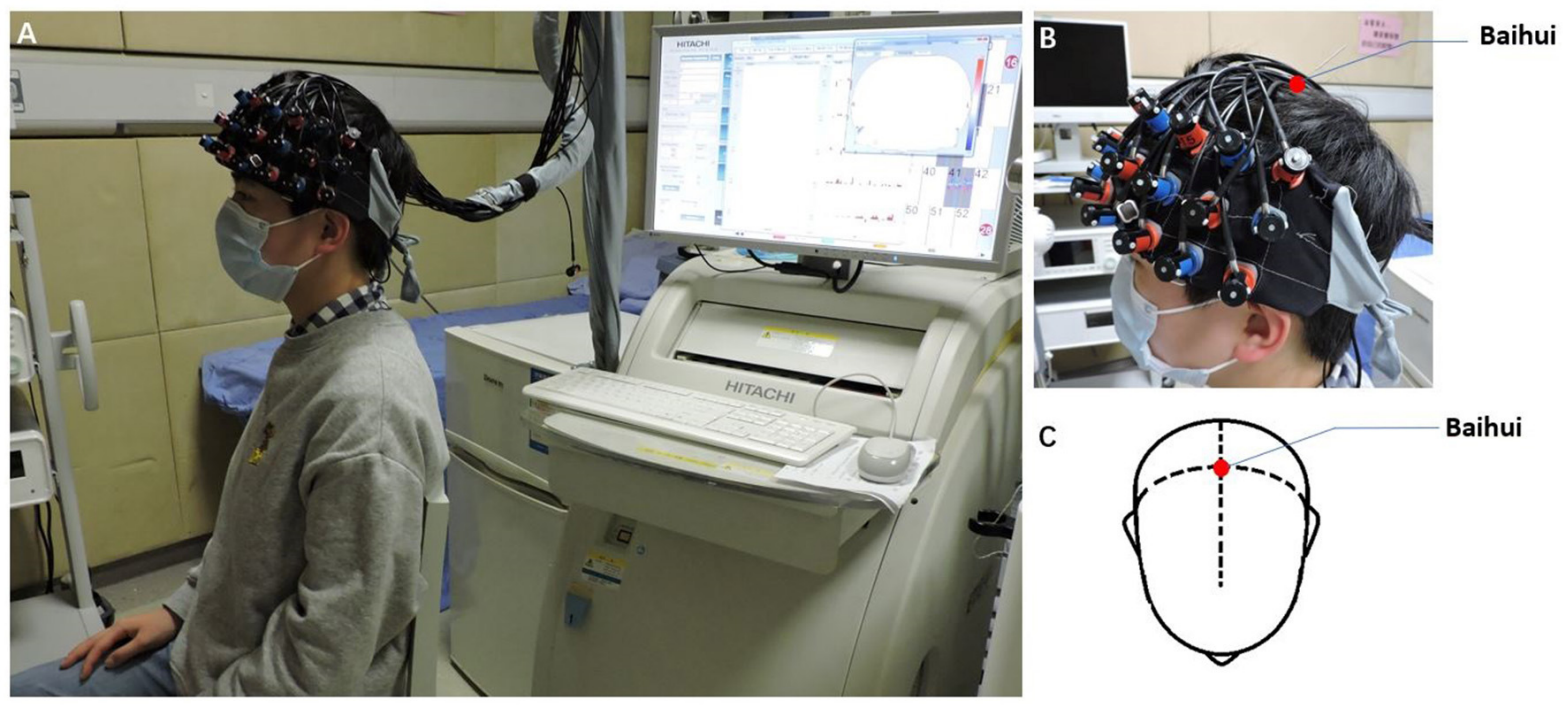
Performing acupuncture. **(A)** Patients were asked to sit and relax on the seat in front of a near-infrared spectroscopy instrument. **(B,C)** The Baihui acupuncture point is shown.

During each 20-s task period, one of three Chinese characters (i.e., “白,” “河,” and “中,” which mean “white,” “river,” and “middle,” respectively) was given as an auditory cue from the control computer place behind a patient. Patients were asked to generate as many words as possible that start with the given character. When the given character was “白,” patients could generate words such as “白天” (daytime), “白领” (white-collar), or “白菜” (Chinese cabbage). The Chinese characters used in the pre-acu and post-acu VFT were different to avoid practice effects. During the task, patients were asked to sit down and relax in front of an fNIRS instrument (Figure 2A) and keep their heads and bodies as motionless as possible.

#### Acupuncture Manipulations

The acupuncture procedure used in the present study was the same as that described in the study by Fernandez Rojas et al. and consisted of three phases of acupuncture manipulations: 5-s needle insertion, three 10-s needle twirls, and 5-s needle removal, interleaved with 30-s resting-state periods (12). The acupoint Baihui, which was used in the study (Figures 2B,C), is located at the intersection point of the midline and the line connecting the ear lobes (19). A qualified TCM acupuncturist performed all these acupuncture manipulations. Patients were asked to sit and relax with their eyes closed and hands resting on their laps during the manipulations.

### fNIRS Data Acquisition

**Figure 3:**
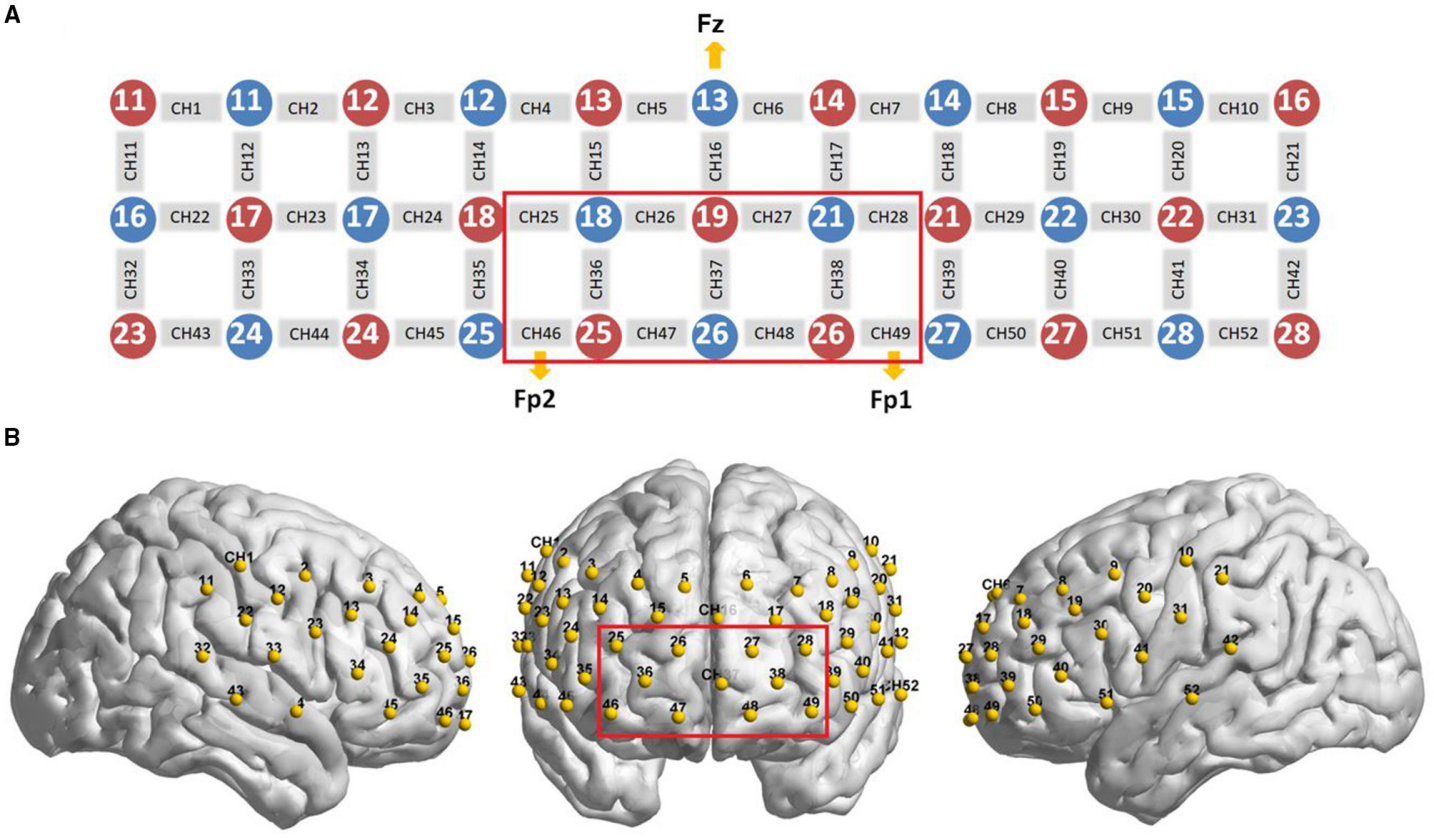
Optode probe set. **(A)** A “3 × 11” measurement patch set with 17 emitter and 16 detector probes positioned alternatively is shown; when the detector number 13 was located at Fz, channels 49 and 46 were placed in FP1 and FP2, respectively. **(B)** The optode probe was placed on the pre-frontal and temporal cortex. The red frame denotes regions of interest.

We used a 52-channel continuous-wave NIRS instrument (ETG-4100 Optical Topography System; Hitachi Medical Co., Japan) with a temporal resolution of 10 Hz to collect imaging data during the two VFTs. The system is equipped with 17 emitters that emit laser lights at two wavelengths (695 and 830 nm) and 16 detectors that detect the corresponding lights after absorption and scattering. A “3 × 11” measurement patch set with the emitter and detector probes positioned alternatingly at 3-cm intervals (Figure 3A) was attached to a nylon cap with several elastic straps used to guarantee good contact to the heads of patients.

In this experiment, we defined the PFC area consisting of 11 channels (Channels 25/26 27/28/36/37/38/46/47/48/49, covering Brodmann 10/11/46/47, which is indicated by a red box in Figure 3) as a region of interest (ROI), in line with other previous fNIRS studies (18, 20). For consistency of measurement patch placement across patients, the international 10–20 system (21) was used to standardize the placement of the probes. Specifically, the detector number 13 was located at Fz, and simultaneously, channels 49 and 46 were placed in FP1 and FP2, respectively (Figure 3A).

Next, a three-dimensional probe position unit for the optical topography system, EZT-DM401 (Hitachi Medical Corporation, Japan), was used to sequentially measure the anatomical landmarks on the heads of patients (nasion, the pre-auricular points anterior to the left and right ears, inion, and Cz) and the probe positions of the optical topography system in the real-world (RW) stereotaxic coordinate system. Subsequently, the RW spatial location data were converted to obtain the cortical positions of 52 channels on the standard Montreal Neurological Institute (MNI) template using the toolbox of NFRI functions (22) available in NIRS-SPM v4.1. The 52-channel patch covered the PFC (Figure 2B) including the ROI defined in this study.

### Statistical Analysis of fNIRS Data

The two toolboxes HOMER2 (23) and NIRS-SPM v4.1 (24) were used in the quantitative analysis of NIRS signals and to calculate the activation maps of oxy-hemoglobin (oxy-Hb) levels. We decided to use oxy-Hb rather than deoxy-hemoglobin (deoxy-Hb) levels because the former is thought to be the most sensitive indicator of regional cerebral blood flow (25) and has a close correlation with the blood oxygen level-dependent response (26, 27).

At first, noisy channels were identified and pruned from the measurement list using the HOMER2 function *hmrPruneChannels* (dRange = 10^3^-10^7^ and *SNRthresh* = 5) (28). Next, optical density (OD) was transferred from optical intensity using the HOMER2 function *hmrintensity2OD*. OD is defined as the logarithmic intensity ratio of the light falling on the material to that of the light transmitted through the material (29).

The channel-wise motion artifacts were then detected by the HOMER2 function *hmrMotionArtifactByChannel*, which was applied to detect the time point where the optical signal was greater than AMPthresh (=5.00, amplitude threshold) or STDEVthresh (=10.00, standard deviation threshold) over the time interval tMotion (=3.0, a predefined time-window) and to mark over ± tMask 0.50 s around that time point as a motion artifact. Channels with too many motion artifacts were rejected.

Subsequently, OD was converted into the concentration changes of oxy-Hb with the modified Beer–Lambert law (30) using NIRS-SPM v4.1. Next, we executed an effective method, wavelet minimum description length (Wavelet-MDL), which is a detrending algorithm to overcome the problem of the presence of an unknown global trend due to breathing, cardiac, vasomotion, or other experimental errors (31). A hemodynamic response function (HRF) filter was then adopted for temporal smoothing to swamp the intrinsic temporal correlations and attenuate high-frequency components (32).

A general linear model (GLM) approach, which has been described extensively in the fNIRS literature (33–35), was then applied to analyze the fNIRS time series in our study. The experimental design matrix for the GLM analysis included two predictors representing boxcar functions corresponding to onsets of word-generating blocks and post-task baseline convolved with an HRF. Word-generating β-values, one of the GLM's weights, representing the oxy-Hb level changes in word-generating stages, and post-task β-values, representing the oxy-Hb level changes in the post-task period, were computed at each channel. Then we defined task-related β-values as word-generating β-values minus post-task β-values.

Afterward, a one-sample *t*-test with the false discovery rate (FDR) method (36) (at a corrected *p* level < 0.05) was conducted on task-related β-values at each channel. Images were created using the *nirs2img* function in the xjview toolbox (https://www.alivelearn.net/xjview/xjView%208%20Manual.pdf), in which the *t*-values of each channel with their corresponding MNI coordinates were converted to an image file. Next, the transformed image files with linear interpolated *t*-values were rendered over a standardized brain model using a BrainNet Viewer toolbox (http://www.nitrc.org/projects/bnv/) (37). A paired two-sample *t*-test was conducted to compare task-related β-values between the post-acu and pre-acu VFTs (β_*post*_ vs. β_*pre*_) at the channels that were significant during the one-sample *t*-test.

Based on the knowledge regarding the differential neuroplastic responses in patients with mild to moderate depression compared to those with severe depression, we repeated the analyses in two separate groups: Group 1 (mild to moderate MDD, HAMD scores of ≤ 35) and Group 2 (severe MDD, HAMD scores of >35) (38). A two-way repeated measures analysis of variance (ANOVA), with time (pre- and post-acu) as the within-subject factor and group (mild to moderate and severe depression) as the between-subject factor, of task-related β-values was performed for all significant channels with *p* < 0.05. A significant main effect or interaction effect was followed with simple effect analyses. With the FDR correction, Pearson's correlation coefficients were calculated between the HAMD scores and VFT-related Δβ (β_*post*−_β_*pre*_) at the significantly activated channels.

In case any significant between-group (i.e., severity group) difference in baseline characteristics was found, a two-way repeated measures analysis of covariance (ANCOVA) would also be performed with the covariate on task-related β-values for all significant channels. Significant group main effect or interaction effect of group by time was followed up with simple effect analysis including the covariate.

## Results

### Cortical Activation During the Pre-acu and Post-acu VFTs

**Figure 4:**
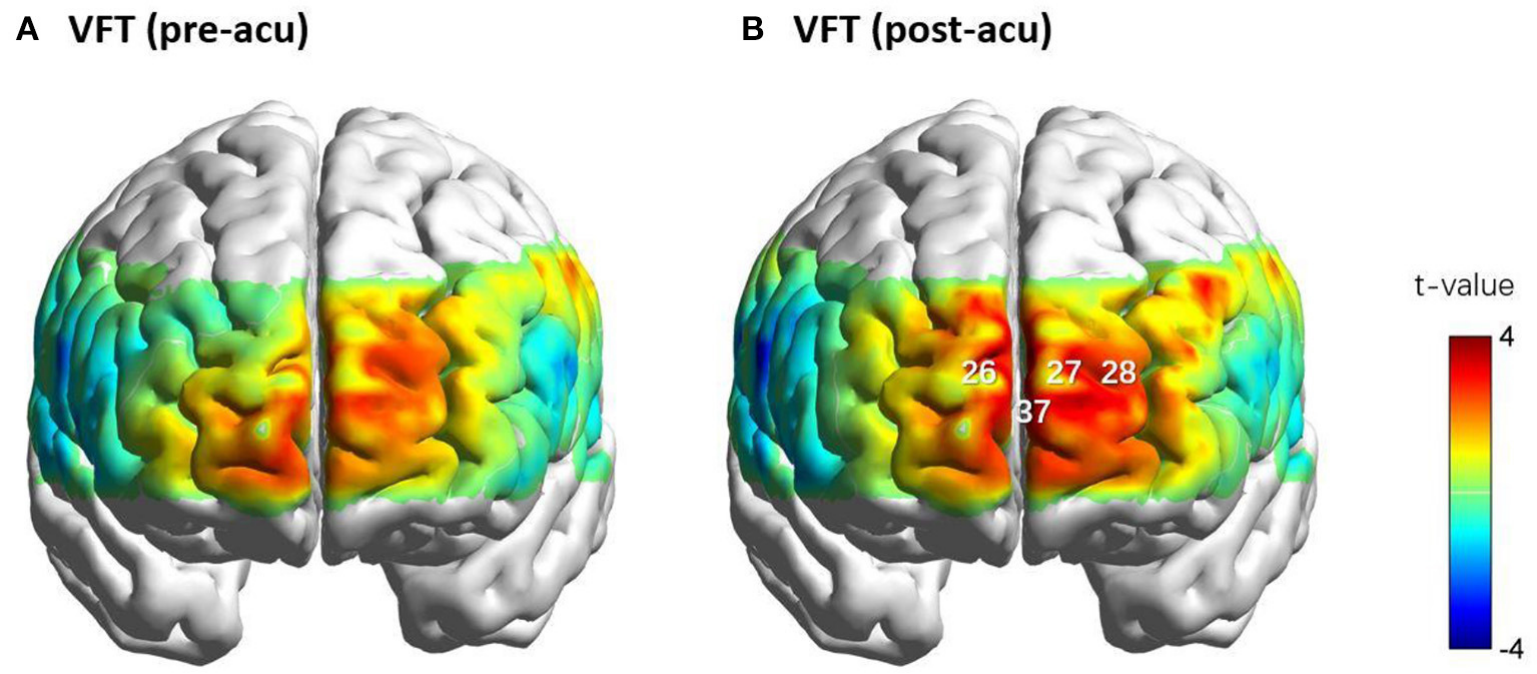
Cortical activation maps of oxy-Hb level during the VFTs before **(A)** and after acupuncture **(B)**. Oxy-Hb level changes in CHs 26, 27, 28, and 37 during the VFTs before and after acupuncture. CHs 26, 28, and 37 were located in the frontopolar area, and CH 28 was located in the left dorsolateral pre-frontal cortex. CH, channel; oxy-Hb, oxy-hemoglobin; VFT, verbal fluency task.

The changes in the oxy-Hb level during the post-acu VFT significantly increased in the frontopolar area (channels 26, 27, and 37) and dorsolateral PFC (DLPFC, channel 28) compared to that in the post-task baseline (FDR-corrected *p* = 0.017, 0.008, 0.036, and 0.021, respectively). Nevertheless, a significant activation of oxy-Hb level changes was not found at any channel during the pre-acu VFT. However, paired *t*-tests showed that task-related β-values during the post-acu VFT (β_post_) were higher than those during the pre-acu VFT (β_pre_) at these channels but did not reach statistical significance (*p* > 0.05). The activation maps for oxy-Hb level changes during the pre-acu and post-acu VFTs are shown in Figure 4.

**Figure 5:**
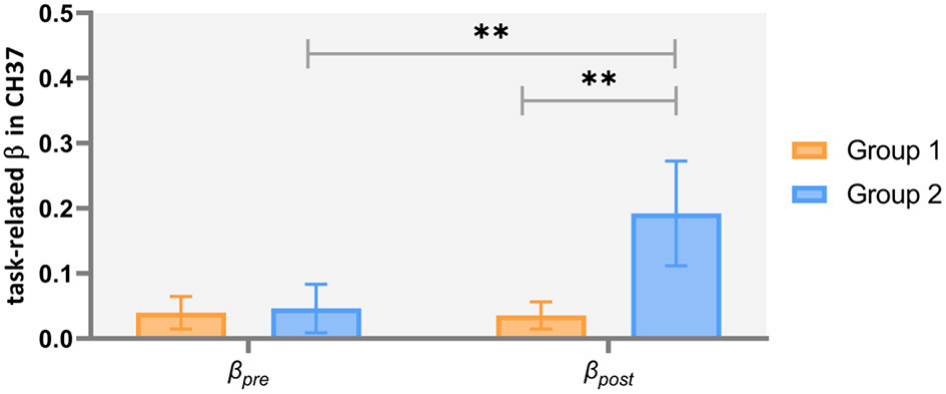
Task-related brain activation in channel 37 in patients with mild to moderate depression (Group 1) and severe depression (Group 2). **p < 0.01.

Two-way repeated ANCOVAs were performed separately on the task-related β-values in channels 26, 27, 28, and 37. In channel 37, we found non-significant covariate-by-group interaction [*F*_(1, 43)_ = 0.976, *p* = 0.329, $\eta_{partial}^{2}$= 0.022], indicating that the regression slopes for the covariate did not differ between both groups, as well as a significant main effect of group [*F*_(1, 44)_ = 5.302, *p* = 0.026, $\eta_{partial}^{2}$= 0.108] and a significant time by group interaction effect [*F*_(1, 44)_ = 5.759, *p* = 0.021, $\eta_{partial}^{2}$= 0.116]. Simple effect analysis showed that, in Group 2 (patients with severe symptoms), the post-acu task-related β-values were higher than the pre-acu values [*F*_(1, 44)_ = 7.945, *p* = 0.007], and during the post-acu VFT, the task-related β-values in Group 2 were higher than those in Group 1 (patients with mild to moderate symptoms) [*F*_(1, 44)_ = 8.145, *p* = 0.007] (Figure 5). There was no significant main effect or interaction effect in other channels (*p* > 0.05).

A two-way repeated measures ANOVA with time as the within-subject factor and group as the between-subject factor on task-related β-values was also conducted considering that the age differences between the groups might not be random (see SI text and Supplementary Figure S1 for details in Supplementary Materials) and yielded the same results as the ANCOVA.

In view of channels 26, 27, and 37 forming a cluster, and all were located in the frontopolar area, we used the averaged data from the three channels in the analysis of ROI 1 and conducted a two-way repeated measures ANOVA as well as ANCOVA on this area. Similar results were found (see SII text and Supplementary Figure S2 for details in Supplementary Materials).

### Correlations Between HAMD Scores and Cortical Activation

**Figure 6:**
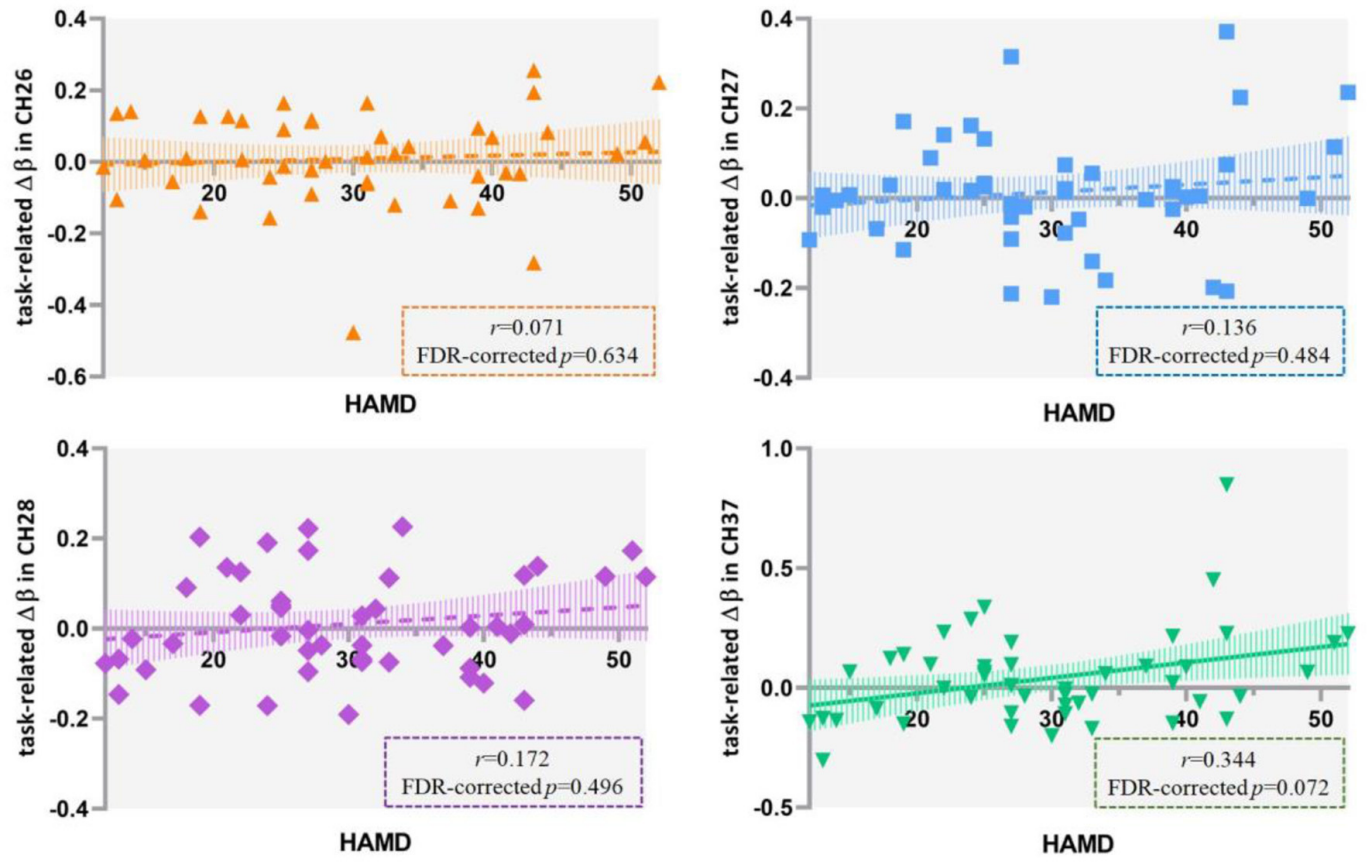
Correlation between HAMD scores and the VFT-related Δβ (β_*post*−_β_*pre*_) in 47 patients with major depressive disorder. HAMD, Hamilton Depression Rating Scale-24; VFT, verbal fluency task.

**Figure 7:**
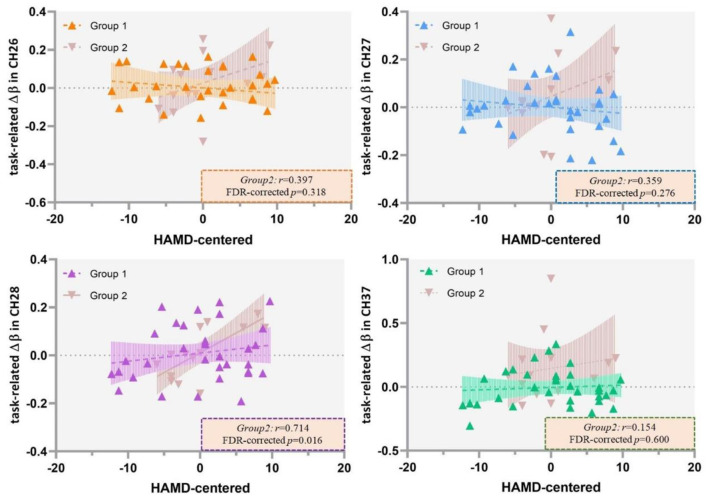
Correlation between HAMD mean centered scores and the VFT-related Δβ (β_*post*−_β_*pre*_) in patients with mild to moderate depression (Group 1) and severe depression (Group 2). HAMD, Hamilton Depression Rating Scale-24; VFT, verbal fluency task.

The results of Pearson's correlation analysis showed that HAMD scores were positively correlated with the VFT-related Δβ (β_post−_β_pre_) in channel 37 at a marginally significant level (*r* = 0.344, FDR-corrected *p* = 0.072) in all the included patients (Figure 6), as well as in ROI 1 (*r* = 0.255, *p* = 0.087), but not in single-channel 26, 27, or 28 (FDR-corrected *p* > 0.05). Among the patients with severe depression as seen in Figure 7, there was a positive correlation between HAMD mean-centered scores and Δβ in channel 28 (*r* = 0.714, FDR-corrected *p* = 0.016). However, the correlation was not found in patients with mild to moderate depression (FDR-corrected *p* > 0.05).

## Discussion

We investigated the PFC activation in response to a single session acupuncture therapy in the Baihui acupoint among a group of patients with MDD. A single session of acupuncture did not significantly increase the activation of the left PFC areas during executive functioning in patients with MDD with mild to moderate symptoms, but it tended to increase the activation of frontopolar areas in patients with severe symptoms. Additionally, a significant correlation between the severity of depressive symptoms and task-related left DLPFC activation by acupuncture was found in patients with MDD with severe symptoms.

The results of this experiment reveal that the activation of the PFC by acupuncture might be modulated by the severity of MDD. Antidepressant medication is the gold standard for the treatment of depression, and its mechanism of action has been extensively studied. According to previous literature, the treatment response to antidepressant medication could be predicted by the initial depression severity: that is, the benefits from antidepressant medications increase with the severity of depression symptoms (4). The antidepressant effect of medications is closely linked with neural activation, specifically in the PFC (39). Many researchers hypothesize that the therapeutic effect of acupuncture on depression may be, at least in part, attributed to the modulation of the PFC. Although the pathogenesis of MDD has not been fully elucidated, there is evidence indicating structural and functional changes in the PFC of patients with MDD (1). A previous fNIRS experiment showed that enhanced activation in the frontopolar areas was associated with the improvement of depressive symptoms in a group of adolescents with depression (40), indicating that the activation of the frontopolar areas is correlated with antidepressant treatment response. The left DLPFC is the most widely studied area in patients with MDD. The imbalance between the left and right DLPFCs (hypoactive left and hyperactive right in patients with MDD) has been linked to negative emotional bias, which is proposed to be a possible behavioral basis for the development of depressive symptoms (41). The studies with antidepressant medications have thus indicated a correlation between the activation of the left DLPFC and treatment-elicited reduction in depressive symptoms. The activations of the DLPFC and frontopolar areas are of importance in the treatment response to acupuncture. Additionally, further studies should investigate whether antidepressant medications and acupuncture share a common therapeutic mechanism that modulates the PFC.

Furthermore, we found a weak correlation between the neuroplastic response to acupuncture in the left frontopolar area and depressive symptoms scores, which suggests that the severity of the disease may be associated with the modulatory effect of acupuncture on the PFC. This finding is similar to that of a meta-analysis in which the response to antidepressant medications was positively correlated with the baseline severity of depression (4). No such relationship was found in patients with mild and moderate depression. However, a strong positive relationship between the neuroplastic response to acupuncture in the left DLPFC and depressive symptoms scores was observed in this study. Differential effects of treatments for depression have been reported in several previous studies with varying results. A study reported that patients with a high baseline depression severity were unlikely to respond to venlafaxine (42). However, another report showed that patients with severe depression had a similar response slope to that of patients with mild to moderate depression after receiving nefazodone and cognitive behavioral therapy (43). These findings indicate a complex interaction between treatment response and severity of depression, and it is likely that the PFC plays a role in mediating this relationship. To the best of our knowledge, no study has explored the interaction between treatment response to acupuncture, the activation of the PFC, and baseline severity in depression. Although we did not have a clinical outcome, our NIRS data suggested that the patients' selection in acupuncture trials needs to be specific as the baseline severity is likely to influence the treatment response, in view of the potential association between the activation of the PFC area and the treatment response to acupuncture.

Although our sample was limited to young and middle-aged adults, age differences were observed between the disease severity groups. Therefore, we conducted an ANCOVA to account for the potentially confounding effect of age. However, we need to acknowledge that the method is unlikely to fully rule out the effect of the covariable (44). Previous studies have reported an age-related decline in PFC function, although the comparison was mostly made between younger adults and older (>65 years) (45, 46). In addition, the age-related effects on neural activation in response to acupuncture are largely unknown. Future studies need to employ age-matched patient groups with different severities of depressive symptoms to verify the present findings.

## Limitations

The current study had some limitations. First, a sham acupuncture control was not employed in this preliminary investigation because a large sample is required for such a complex study design. Based on the findings from this experiment, further studies should be conducted with a larger sample and sham acupoint controls. Second, we only selected a single acupoint for the treatment. This is in contrast to clinical acupuncture therapy where multiple acupoints are selected. Third, we did not enroll healthy people as controls to investigate whether a unique activation pattern in patients with depression might exist. Finally, our analysis on severity of depression might have been affected by unequal numbers of patients with mild to moderate and severe depression; therefore, these results should be considered preliminary.

## Conclusion

A single session of acupuncture could not significantly modulate the activities of the PFC in patients with mild to moderate depressive symptoms, but it tended to enhance the activation of the frontopolar area in patients with severe symptoms. Among patients with severe depression, there was a correlation between the activation by acupuncture of left dorsolateral PFC during executive functioning and the severity of depressive symptoms, suggesting that the brain activity induced by acupuncture is likely to be influenced by the baseline disease severity in patients with MDD. The results also highlighted the potential importance of patient selection, in terms of baseline severity of depressive symptoms, when conducting clinical trials using acupuncture in depression treatment.

## Data Availability Statement

The raw data supporting the conclusions of this article will be made available by the authors, without undue reservation.

## Ethics Statement

The study was approved by the Human Research Ethics Committee of West China Hospital (Ethical approval number: WCH201801201134). The patients/participants provided their written informed consent to participate in this study.

## Author Contributions

PW and ZZ conceived and designed the experiments. JH performed the experiments. TZ analyzed the neuroimaging and behavioral data. TZ and JZ drafted the manuscript. PW, ZZ, TZ, and JZ reviewed the manuscript. All authors contributed to the article and approved the submitted version.

## Conflict of Interest

The authors declare that the research was conducted in the absence of any commercial or financial relationships that could be construed as a potential conflict of interest.

## Publisher's Note

All claims expressed in this article are solely those of the authors and do not necessarily represent those of their affiliated organizations, or those of the publisher, the editors and the reviewers. Any product that may be evaluated in this article, or claim that may be made by its manufacturer, is not guaranteed or endorsed by the publisher.
