# Supplementary material for: Neural Activation via Acupuncture in Patients With Major Depressive Disorder: A Functional Near-Infrared Spectroscopy Study
Source: Front Psychiatry. 2021 Nov 12;12:669533. doi: 10.3389/fpsyt.2021.669533 (PMC8632864; doi:10.3389/fpsyt.2021.669533)
Supplement: Supplementary file 1 [file Data_Sheet_1.docx]

**Supplementary Material**

**Neural Activation to Acupuncture in Patients with Major Depression Disorders: A Functional Near-infrared Spectroscopy Experiment**

Tingyu ZHANG^a*^, Jiaqi ZHANG^b*^, Jiaxi HUANG^c^, Zhong ZHENG^c#^ and Pu WANG^a,d#^

^a^Department of Rehabilitation Medicine, The seventh Affiliated Hospital Sun Yat-sen University, Shenzhen, China;

^b^Department of Rehabilitation Sciences, The Hong Kong Polytechnic University, Hong Kong SAR, China;

^c^ Mental Health Center, West China Hospital/West China School of Medicine, Sichuan University, Chengdu, China

^d^ Guangdong Engineering and Technology Research Center for Rehabilitation Medicine and Translation, Guangzhou, China

^*^ These authors contributed equally to this work.

| **Content** | **Page** |
| --- | --- |
| SI text | 2 |
| Figure S1 | 3 |
| SII text | 4 |
| Figure S2 | 5 |

**SI text**

Two-way ANOVAs were performed separately on the task-related *β*-values in CH26, CH27, CH28, and CH37. There was a significant main time effect (*F*(1,45) =5.736, *p*=0.021, *η^2^_partial_*=0.113), a marginal significant group effect (*F*(1,45) =3.320, *p*=0.075, *η^2^_partial_*=0.069) and a significant time by group interaction effect at CH37 (*F*(1,45) =6.459, *p*=0.015, *η^2^_partial_* =0.126). Simple effect analysis showed that, in Group 2, the task-related *β*-values after the acupuncture manipulation was higher than that before the acupuncture manipulation (*F*(1,45) =8.677, *p*=0.005), and during the VFT-task after the acupuncture manipulation, the task-related *β*-values in Group 2 was higher than that in Group 1 (*F*(1,45) =6.641, *p*=0.013) (see Figure 4). There was no significant main effect or interaction effect in other channels (*ps* > 0.05).


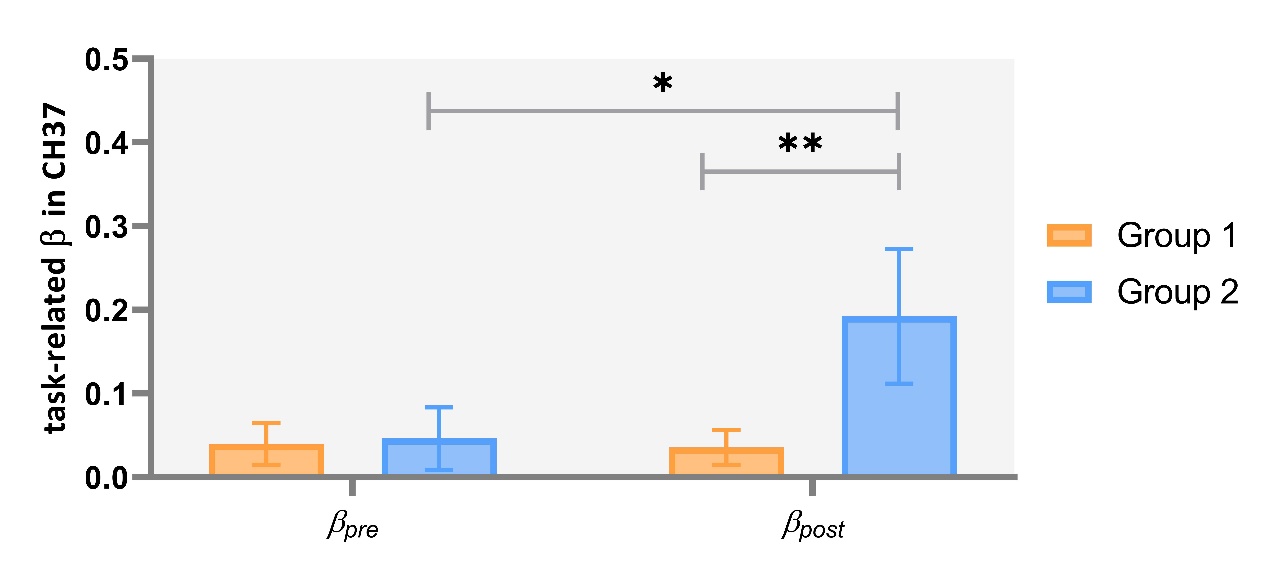


**FIGURE S1.** ANOVA results. Task-related brain activation in Channel 37 in patients with mild to moderate depression (Group 1) and severe depression (Group 2). . **p*<0.05; ***p*<0.01

**S****Ⅱ text**

Two-way ANOVAs were contacted on the task-related *β*-values in ROI1 (CH27, CH28, and CH37 which were located in the frontopolar area). There was a marginal significant main time effect (*F*(1,45) 3.290, *p*=0.076, *η^2^_partial_*=0.068) and a marginal significant time by group interaction effect (*F*(1,45) =3.529, *p*=0.067, *η^2^_partial_* =0.073).

Two-way repeated ANCOVAs were also performed on the task-related *β*-values in ROI1. There was a marginal significant covariate-by-group interaction (*F*[1,43]=0.548, *p*=0.436, *η2_partial_*=0.013), which indicated that the regression slopes for the covariate did not differ between both groups. We also found a marginal significant main effect of group (*F*[1,44]=3.352, *p*=0.074, *η2_partial_*=0.071) and a marginal significant time by group interaction effect (*F*[1,44]=3.735, *p*=0.060, *η2_partial_*=0.078). Simple effect analysis showed that, in Group 2 (patients with severe symptoms), the post-acu task-related β-values were higher than the pre-acu values (*F*[1,44]=5.039, *p*=0.030), and during the post-acu VFT, the task-related β-values in Group 2 were higher than those in Group 1 (patients with mild to moderate symptoms) (*F*[1,44]=5.809, *p*=0.020) (Figure S2).


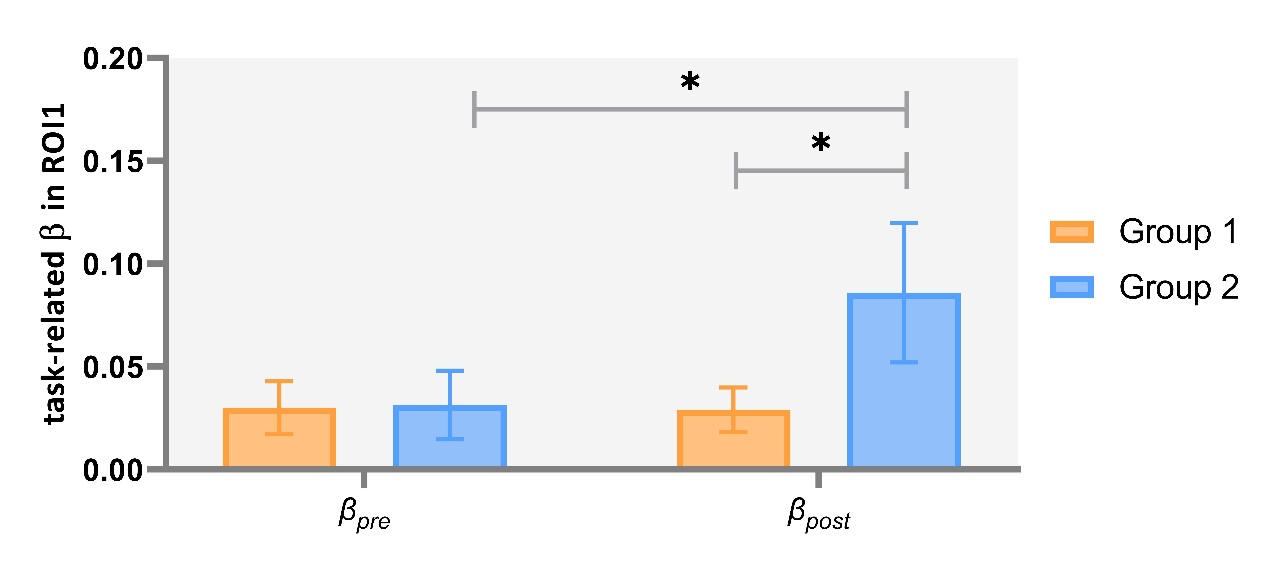


**FIGURE S2.** ANCOVA results. Task-related brain activation in ROI 1 in patients with mild to moderate depression (Group 1) and severe depression (Group 2). **p*<0.05.
